# Clomiphene Citrate Administered in Periconception Phase Causes Fetal Loss and Developmental Impairment in Mice

**DOI:** 10.1210/endocr/bqae047

**Published:** 2024-04-12

**Authors:** Peck Y Chin, Hon Yeung Chan, Tom E C Kieffer, Jelmer R Prins, Darryl L Russell, Michael J Davies, Sarah A Robertson

**Affiliations:** Robinson Research Institute and School of Biomedicine, University of Adelaide, Adelaide, SA 5005, Australia; Robinson Research Institute and School of Biomedicine, University of Adelaide, Adelaide, SA 5005, Australia; Robinson Research Institute and School of Biomedicine, University of Adelaide, Adelaide, SA 5005, Australia; Department of Obstetrics and Gynecology, University Medical Center Groningen, University of Groningen, 9713 GZ Groningen, The Netherlands; Amsterdam University Medical Center, Meibergdreef 9, 1105 AZ Amsterdam, The Netherlands; Department of Obstetrics and Gynecology, University Medical Center Groningen, University of Groningen, 9713 GZ Groningen, The Netherlands; Robinson Research Institute and School of Biomedicine, University of Adelaide, Adelaide, SA 5005, Australia; Robinson Research Institute and School of Biomedicine, University of Adelaide, Adelaide, SA 5005, Australia; Robinson Research Institute and School of Biomedicine, University of Adelaide, Adelaide, SA 5005, Australia

**Keywords:** clomiphene citrate, infertility, uterus, pregnancy, fetal growth, mice

## Abstract

Clomiphene citrate is a common treatment for ovulation induction in subfertile women, but its use is associated with elevated risk of adverse perinatal outcomes and birth defects. To investigate the biological plausibility of a causal relationship, this study investigated the consequences in mice for fetal development and pregnancy outcome of periconception clomiphene citrate administration at doses approximating human exposures. A dose-dependent adverse effect of clomiphene citrate given twice in the 36 hours after mating was seen, with a moderate dose of 0.75 mg/kg sufficient to cause altered reproductive outcomes in 3 independent cohorts. Viable pregnancy was reduced by 30%, late gestation fetal weight was reduced by 16%, and ∼30% of fetuses exhibited delayed development and/or congenital abnormalities not seen in control dams, including defects of the lung, kidney, liver, eye, skin, limbs, and umbilicus. Clomiphene citrate also caused a 30-hour average delay in time of birth, and elevated rate of pup death in the early postnatal phase. In surviving offspring, growth trajectory tracking and body morphometry analysis at 20 weeks of age showed postweaning growth and development similar to controls. A dysregulated inflammatory response in the endometrium was observed and may contribute to the underlying pathophysiological mechanism. These results demonstrate that in utero exposure to clomiphene citrate during early pregnancy can compromise implantation and impact fetal growth and development, causing adverse perinatal outcomes. The findings raise the prospect of similar iatrogenic effects in women where clomiphene citrate may be present in the periconception phase unless its use is well-supervised.

Clomiphene citrate is a triphenylethylene derivative and first-generation selective estrogen receptor modulator that has been utilized extensively as a first-line treatment for ovulation induction in anovulatory subfertile women for 50 years. It is listed as an essential drug by the World Health Organization, and is generally effective and low cost (1, 2). Clomiphene citrate was developed in the 1950s following efforts to formulate new nonsteroidal contraceptive compounds. One of several candidate compounds, clomiphene citrate (known then as MRL/41) unexpectedly acted to compete with estradiol for estrogen receptors to elicit mixed estrogen agonist/estrogen antagonist activity. Clinical studies then revealed its capacity to induce ovulation in amenorrheic women (3), and its potential for widespread use in subfertile women was recognized (4).

The current standard practice is to administer clomiphene citrate for 5 days each menstrual cycle, commencing on cycle days 2 to 5. Clomiphene doses start at 50 mg/day and may progressively increase to 250 mg/day (5, 6), although a dose above 100 mg/day is not recommended by the US Food and Drug Administration (7). The current guidelines of the National Institute for Health and Care Excellence recommend use of clomiphene citrate for 6 ovulatory cycles (8) and no more than 12 cycles (9-11), as prolonged use is considered to increase the risk of ovarian cancer (10, 12). This protocol is thought to achieve ovulation enhancement by acting in the hypothalamus and pituitary to block negative feedback effects of circulating estradiol, preventing estrogen-mediated suppression of follicle-stimulating hormone release, and thus promoting growth of preovulatory ovarian follicles (13). This mode of action was inferred from studies showing that during clomiphene treatment, the frequency and amplitude of hypothalamic gonadotropin-releasing hormone pulses increase, thereby stimulating the pituitary gland to release more follicle-stimulating hormone and luteinizing hormone, resulting in increased folliculogenesis (5, 13). However, there are unresolved questions regarding the mechanisms of clomiphene actions and their effects (14), since in other studies its antiestrogenic effects can be shown to suppress the luteinizing hormone surge (15), but direct effects on steroidogenic function in granulosa cells during folliculogenesis (16) as well as direct effects in the endometrium (17), also seem likely.

The safety of clomiphene citrate for human fetal development has not been formally assessed in a prospective study. Previous studies have indicated teratogenic effects of clomiphene citrate in humans, including neural tube defects and hypospadias (18-21). Data from a variety of sources including adverse event reports, case reports, cohort studies, and case–control studies link clomiphene citrate use to major birth defects including blindness, limb reduction, cardiac, renal and anal agenesis, and urogenital defects (22, 23). Sporadic adverse maternal reactions in humans have been noted, including ovarian hyperstimulation syndrome, visual distortion, and, in rare cases, blindness and death. Clomiphene citrate is also associated with miscarriage, low birth weight, and premature birth (24, 25). However, it has been argued that the evidence for a causal effect is unclear and inconsistent (17). Importantly, because these observations are made in women with clinical subfertility, the extent to which the drug as opposed to the underlying clinical condition is causal remains unknown, and is a constraint in interpreting its potential causal effects.

Teratogenic effects of clomiphene citrate have been demonstrated in animal studies, including fetal abnormalities such as exencephaly, cataracts, cleft palette, and other malformations in rats and mice (26-28). These studies have limitations in their relevance to human clomiphene citrate usage, particularly with regard to drug dose and timing of administration. The most informative study on effects of periconception exposure comes from Dziadek et al (27), who showed impaired embryo implantation and fetal growth restriction, with occasional neural tube defect, when clomiphene was administered twice at a dose of 2.5 µg/g prior to ovulation or after mating. However, the doses utilized in this study were higher than might occur in many women, and the profound implantation failure observed following periconception administration precluded evaluation of effects on fetal development specific to this time window. Additionally, the dosing schedule commenced prior to ovulation for mice exposed in the periconception phase, so effects mediated via oocyte quality could not be ruled out.

It is therefore imperative to expand understanding of effects of clomiphene citrate on fetal development in animal models, where effects of the drug can be investigated independent of indications for infertility, and dosing protocols can be designed to investigate possible mechanisms of action. Here, we examine the effects of low-dose clomiphene citrate in the periconception phase on pregnancy outcomes and the consequences of clomiphene citrate exposure for fetal and postnatal development using a study design to specifically interrogate effects mediated via the embryo and uterus.

## Materials and Methods

### Animals and Mating

CBA × C57Bl/6 F1 (CBA F1) female mice and BALB/c male mice were purchased from the University of Adelaide Animal Facility. Adult naturally cycling females (8-12 weeks) were housed with a proven fertile stud male (2:1 female to male ratio) and checked each morning between 09:00 and 11:00 hours for the presence of a vaginal copulatory plug. The day of sighting of a vaginal plug was designated gestational day (GD) 0.5 and mated females were separated from males. All mice were housed under specific pathogen-free conditions on a 12 light:12 dark cycle, and food and water were administered ad libitum. All experiments were performed in accordance with the Australian Code of Practice for the Care and Use of Animals for Scientific purposes, with approval of the University of Adelaide Ethics Committee (Approval number M-2015-260A).

### Treatment Protocols

Mated females were administered 5 µg, 15 µg, or 50 µg of clomiphene citrate (Sigma) (0.25, 0.75, and 2.5 µg/g) in 100 µL saline subcutaneously, or saline alone, at 10:00 hours to 12:00 hours on GD 0.5 and GD 1.5. In a dose–response experiment, the effect of clomiphene citrate dose on pregnancy parameters at GD 14.5 was evaluated. In additional groups of mated females given the midrange dose of 15 µg of clomiphene citrate or saline control on GD 0.5 and GD 1.5, late gestation outcomes were evaluated at GD 18.5, or dams were allowed to progress to term for analysis of perinatal and offspring outcomes.

In both the GD 14.5 and GD 18.5 cohorts, mice were killed by cervical dislocation at 10:00 hours to 12:00 hours. The uterus was removed and the number of viable, dead, and resorbing implants were counted. Mice were classified as pregnant when at least 1 viable implantation site was seen, and pregnancy rate was calculated as % pregnant dams/mated females. Each viable fetus was dissected from the amniotic sac and umbilical cord, then fetal and placental weights were recorded and the fetal:placental weight ratio was calculated. GD 14.5 fetuses were assessed for normal mouse embryo development using Theiler stage (TS) criteria, and fetal length was determined. GD 18.5 fetuses were fixed in Bouin's solution for morphometric and histological analysis.

### Fetal Morphometric and Histological Analysis

A subset of fetuses excised from dams at GD 18.5, after administration of 15 µg clomiphene citrate or saline control on GD 0.5 and GD 1.5, were analyzed by whole body histology at the Australian Phenomics Network (University of Melbourne, Australia). Analysis was conducted on 30 fetuses (9 male, 21 female) from 13 dams in the clomiphene citrate group, and 5 fetuses (2 male, 3 female) from 5 dams in the control group. After excision, fetuses were immediately fixed in Bouin's solution for 24 hours, then washed and stored in 70% ethanol. Fetuses were processed to cut 5 µm sagittal sections from the midline, lateral sections through the body, and rostral/transverse sections through the head. Slides were stained with hematoxylin and eosin, and sections at 100 µm intervals were examined for structural features of all thoracic and abdominal organs and skeletal tissue, and the nasal/oral region, brain, eyes, and auditory/vestibular apparatus. All slides were reviewed by a veterinary pathologist at the Australian Phenomics Network and scored for structural indications of developmental anomalies by comparison with standard reference materials for mouse fetal development (29, 30).

### Offspring Analysis

Mated females allocated to the term cohort continued pregnancy to term, when the number of pups born, length of gestation, and birth weight were recorded. Pups were weighed at 12 to 24 hours after birth, 8 days, and 21 days, when pups were weaned and housed with littermates according to sex. All progeny were weighed again at 4 weeks and then every 2 weeks until 20 weeks of age.

At weaning, the anogenital distance (AGD, distance from the anus to the genital papilla) was measured in both male and female progeny. Female progeny were checked daily from day 21 of age for the presence of vaginal opening as a measure of puberty onset.

To measure reproductive parameters in male offspring, the epididymis was dissected from male progeny at 20 weeks of age and placed in 1 mL of 37 °C RPMI media before piercing the tissue to allow sperm to diffuse for 10 minutes. Sperm count analysis was conducted by diluting the sperm 1:20 in MilliQ H_2_O. Diluted sperm was then loaded onto a hemocytometer, and number of intact sperm was counted after 4 to 5 minutes. Sperm counts were averaged and total sperm number within the epididymis was calculated using the equation: total sperm = sperm count (average) × dilution factor × 10^4^. To determine the sperm motility profile, 10 µL of undiluted sperm solution was loaded onto a glass slide and motility was categorized as progressive motile, nonprogressive motile, total motile (the sum of progressive and nonprogressive), or immotile. A minimum of 200 sperm was counted before calculating percentages of each category from the total sperm counted, as previously described (31).

### Body Morphometry Analysis

At 20 weeks, male and female progeny were killed by cervical dislocation, weighed, and autopsied for full body composition analysis. The following tissues were excised and weighed individually; brain, heart, lungs (left and right), kidneys (left and right), liver, adrenal glands (left and right), thymus, spleen, testes (males, left and right), seminal vesicles (males, left and right), epididymides (males, left and right), ovaries (females, left and right), uterus (females), quadriceps (left and right), triceps (left and right), biceps (left and right), gastrocnemius muscle (left and right), retroperitoneal fat, perirenal fat, epididymal fat (males, left and right), and parametrial fat (females). Weights of bilateral tissues and organs were combined for each mouse. Total muscle weight was calculated by summing the weights of quadriceps, triceps, and biceps and gastrocnemius muscles. Total central fat weight was calculated by summing the weights of retroperitoneal fat, perirenal fat, and epididymal fat (for males) or parametrial fat (for females), and the muscle:central fat ratio was determined. Total central fat weight was subtracted from total body weight to calculate total lean weight. Results are expressed both as relative weight (normalized to total lean weight) or absolute weight.

### Immunohistochemistry

Uteri were collected from additional groups of unmated estrous CBA F1 mice, or CBA F1 mice mated to BALB/c males. Mated females were administered 50 µg of clomiphene citrate or saline according to the protocol described above, and mice were killed on GD 0.5, GD 1.5, or GD 2.5 (2 hours after most recent clomiphene citrate treatment was administered on GD 0.5 or 1.5). Uteri were embedded in OCT compound (Tissue-Tek, Sakuta Fintek, Torrance, CA) and frozen in liquid nitrogen-cooled isopentane. Cryostat sections (6 µm) were mounted on glass slides and fixed in 96% ethanol (vol/vol) for 10 minutes at 4 °C. For localization of CD11B^+^ macrophages and neutrophils, sections were incubated with rat antimouse CD11B hybridoma supernatant (AB_3095742, M1/70.15.11.5.HL, American Type Culture Collection), followed by biotinylated rabbit antirat IgG (1:600, Vector Laboratories) for 40 minutes at room temperature, and detection with ABC Vectorstain Elite reagents (Vector Laboratories), 3,3′-diaminobezidine (DAB) and H_2_O_2_ (SigmaFast DAB; Sigma). Tissue sections were counterstained with hematoxylin (Sigma). Images of stained sections were captured using a Hamamatsu Photonics Nanozoomer (Hamamatsu, Shizouka, Japan).

Staining was quantified using the video image analysis software, Video Pro (Leading Edge Software, Adelaide, Australia). The area of positive staining was calculated as the area of DAB staining expressed as a percentage of the total stained (hematoxylin + DAB) area. A minimum of 2 nonserial sections were assessed for each sample, and 4 to 6 fields were analyzed per section. To determine the percentage of endogenous peroxidase–positive eosinophils, slides were stained with DAB and hematoxylin only. The value for DAB positivity was subtracted from the total positivity detected for CD11B staining.

### Statistical Analysis

All statistical analysis was conducted using SPSS for Windows, version 20.0 software (SPSS Inc, Chicago, IL). Pregnancy viability was analyzed by the χ^2^ test. Maternal parameters including gestation length and litter size data expressed as mean ± standard error of the mean (SEM) were analyzed by 1-way analysis of variance (ANOVA) and post hoc Sidak t-test. Fetal and offspring parameters including fetal and placental weights, postnatal weights, and body composition data are expressed as estimated marginal means ± SEM and analyzed by mixed model linear repeated measures ANOVA and post hoc least significance difference (LSD) t-test, with mother as subject and litter size as a covariate. Body composition data expressed as relative weight or absolute weight and effect of clomiphene citrate was compared by mixed model linear repeated measures ANOVA and post hoc LSD t-test, with mother as subject and litter size as covariate. Effect of treatment group on uterine CD11B leukocytes was analyzed by 2-way ANOVA. Differences between groups were considered significant when *P <* .05.

## Results

### Clomiphene Citrate Affects Fetal Development at GD 14.5 in a Dose-Dependent Manner

To evaluate the effects of clomiphene citrate present in the periconception phase, mated CBA F1 females were administered clomiphene citrate on GD 0.5 and GD 1.5 at 1 of 3 doses (5 µg, 15 µg, or 50 µg clomiphene citrate). This dose range was chosen to approximate clomiphene citrate levels expected to occur in women who conceive during a clomiphene citrate treatment cycle (50-250 mg/day = 1-5 mg/kg/day in a 50 kg woman). The dosing schedule was chosen to focus evaluation of clomiphene citrate effects in the periconception and peri-implantation phase, without complications relating to ovulation or oocyte quality.

**Figure 1.**
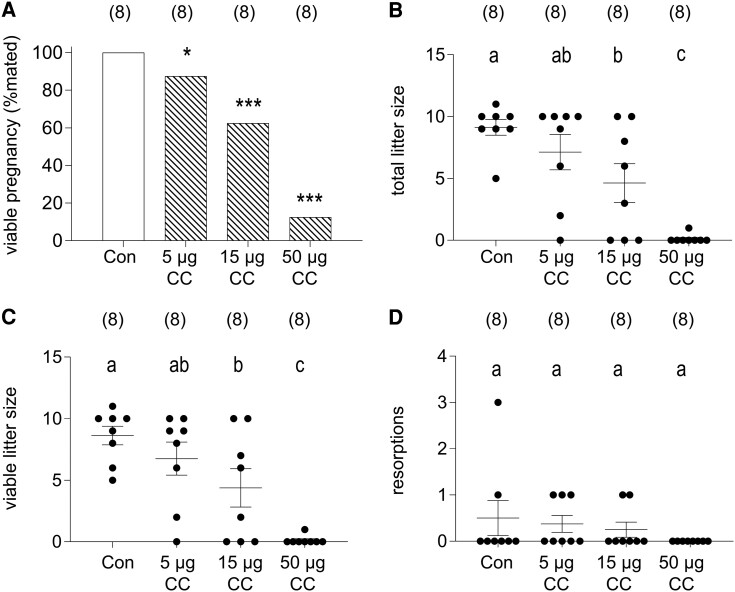
The effects of periconception clomiphene citrate on pregnancy outcomes measured at GD 14.5. CBA F1 females were mated to BALB/c males and administered 5 µg, 15 µg, 50 µg of clomiphene citrate or saline vehicle control subcutaneously on GD 0.5 and GD 1.5. Pregnancy rate is the proportion of mated mice with at least 1 viable fetus on GD 14.5 (A). Other data are total litter size/mated female (B), viable litter size/mated female (C), and number of resorptions/mated female (D). The number of mated dams per group is shown in parentheses. Data in B-D are shown as mean ± SEM with individual dams indicated by symbols. Treatment effects were analyzed by χ^2^ test for pregnancy rate (A, *P < .05, ***P < .0001) and 1-way ANOVA with Sidak post hoc t-test for other parameters (^a,b,c^Different letters indicate differences between groups, *P <* .05).

Mice given clomiphene citrate or saline control in early pregnancy were assessed for fetal outcomes on GD 14.5. Incidence of viable pregnancy (indicated by at least 1 viable fetus) was not affected in the 5 µg clomiphene citrate group (7/8, 87.5%), compared with the saline control group (8/8, 100%) and moderately reduced in the 15 µg clomiphene citrate group (5/8, 62.5%, *P* = .134) (Fig. 1A). In the 50 µg clomiphene citrate group, progression to viable pregnancy was substantially impaired (1/8, 12.5%, *P <* .001) (Fig. 1A).

When pregnancy success was evaluated by total litter size per mated dam, progressively adverse effects of clomiphene citrate were seen. Average litter size declined as clomiphene citrate dose increased, with a 50% and 99% decline at 15 µg and 50 µg, respectively (Fig. 1B, *P* = .037 and *P* < .001). The number of viable implantation sites was reduced as drug dose increased, with a 49% decline at 15 µg (*P* = .049) and a 98% decline at 50 µg (*P* < .0001) (Fig. 1C). Smaller litter sizes appeared to reflect implantation failure or very early fetal loss, as there was no significant increase in visible fetal resorptions in dams given clomiphene citrate (Fig. 1D).

**Figure 2.**
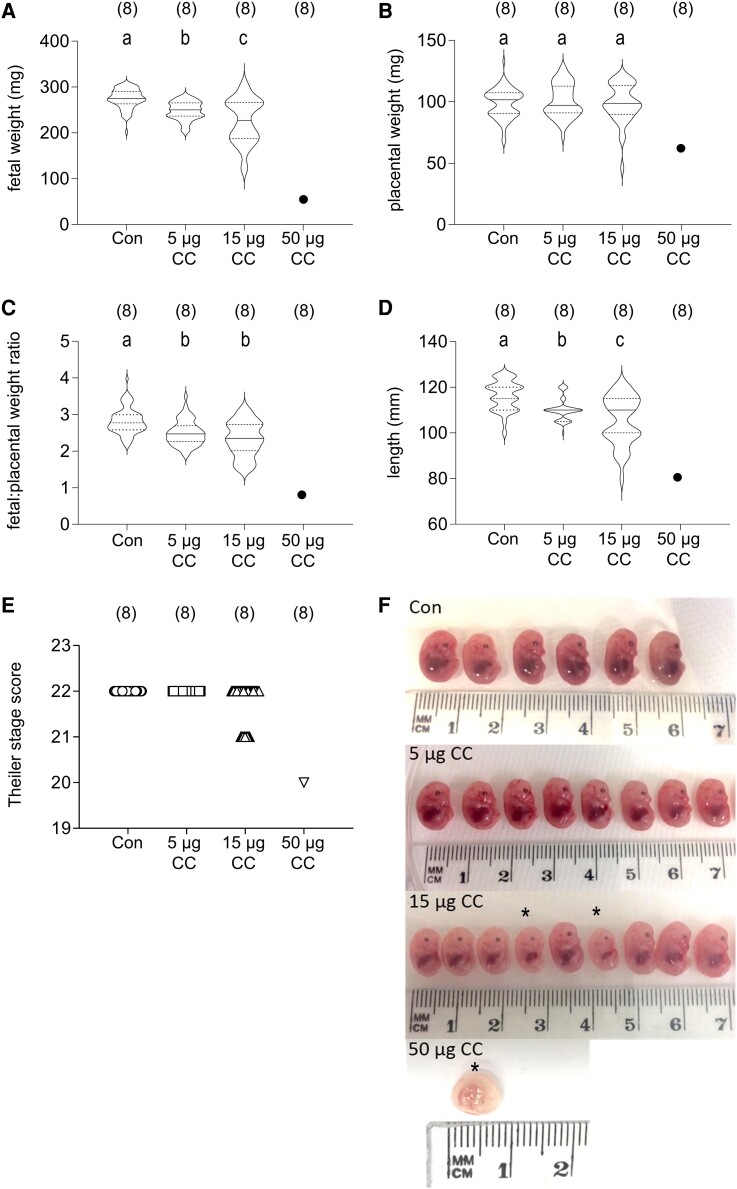
The effects of periconception clomiphene citrate on fetal and placental outcomes analyzed at GD 14.5. CBA F1 females were mated to BALB/c males and administered 15  µg clomiphene citrate or saline control subcutaneously on GD 0.5 and GD 1.5. Data are fetal weight (A), placental weight (B), fetal:placental weight ratio (C), fetal length (D), and Theiler stage score (E). Fetuses dissected from 1 representative dam in each treatment group are shown (F). Clomiphene citrate-exposed fetuses (CC) marked with an asterisk (*) have visibly delayed development compared with control (Con) fetuses and other CC fetuses. The number of mated dams per group is shown in parentheses. Data in A-D are shown as violin plots with median and quartile values marked, and data for individual dams is indicated by symbols in E. In A-D, treatment effects were analyzed by mixed model ANOVA with dam as subject and LSD post hoc t-test (^a,b,c^Different letters indicate differences between groups, *P <* .05).

Fetal weights were reduced by 10% in the 5 µg clomiphene citrate dose and 17% in the 15 µg clomiphene citrate dose compared with control (Fig. 2A, *P <* .0001). Several fetuses in dams given 15 µg of clomiphene were obviously severely growth restricted, and the 1 fetus found in 1 of 8 dams given 50 µg of clomiphene showed profoundly retarded development (Fig. 2F). Placental weight was unaffected in the 5 µg or 15 µg of clomiphene citrate groups compared with control (Fig. 2B). The fetal:placental ratio was reduced by 13% and 15% following treatment with 5 µg and 15 µg of clomiphene citrate (Fig. 2C, *P <* .001). This was accompanied by a 5% and 8% reduction in fetal length in dams in the 5 µg and 15 µg clomiphene citrate groups, respectively, compared with the control dams (Fig. 2D, *P <* .001).

Morphological staging of fetal development using the TS scoring criteria (wherein normal GD 14.5 pups are scored as TS 22) showed no difference in fetal development in dams administered 5 µg of clomiphene citrate. Treatment with 15 µg or 50 µg of clomiphene citrate delayed fetal development as measured by TS in 8/35 and 1/1 fetuses respectively (Fig. 2E and 2F). Fetuses of clomiphene citrate treated dams with delayed development as indicated by TS score had a lower fetal:placental weight ratio than fetuses with on-time development (*P* = .002, Fig. S1) (32).

### Clomiphene Citrate Treatment Delays Fetal Development Evident at GD 18.5

**Figure 3.**
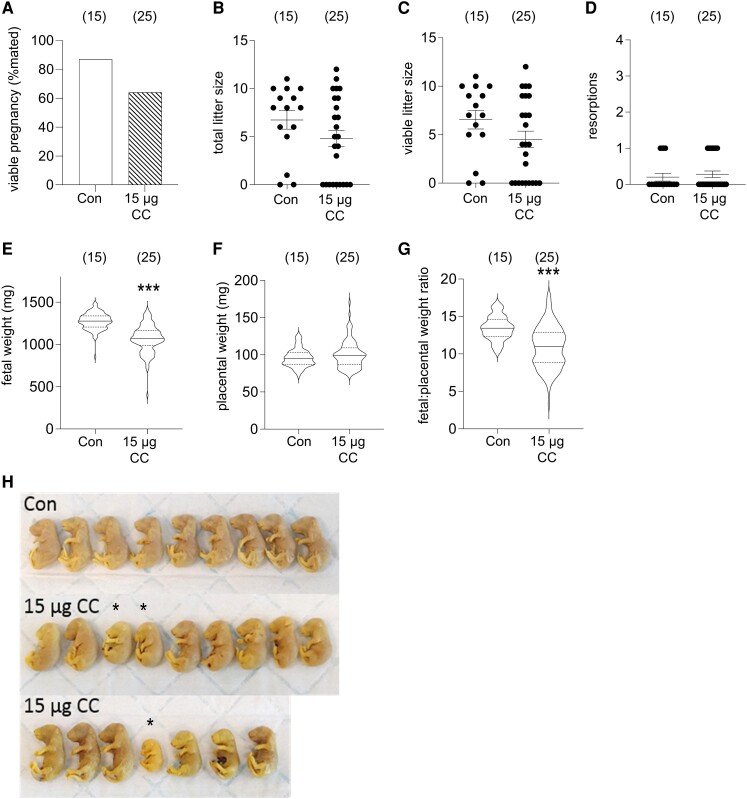
The effects of periconception clomiphene citrate on pregnancy outcomes analyzed on GD 18.5. CBA F1 females were mated to BALB/c males and administered 15 µg of clomiphene citrate or saline control subcutaneously on GD 0.5 and GD 1.5. Pregnancy rate is the proportion of mated mice with at least 1 viable fetus on GD 14.5 (A). Other data are total litter size/mated female (B), viable litter size/mated female (C), and number of resorptions/mated female (D), fetal weight (E), placental weight, and (F) fetal:placental weight ratio (G). Fetuses dissected from representative dams in each treatment group are shown (H). Clomiphene citrate-exposed fetuses (CC) marked with an asterisk (*) have visibly delayed development compared with control (Con) fetuses and other CC fetuses. The number of mated dams per group is shown in parentheses. Data for individual dams is indicated by symbols in B-D. Data in E-G are shown as violin plots with median and quartile values marked. Treatment effects were analyzed by χ^2^-test for pregnancy rate (A) and Sidak t-test for B-D. In E-G, treatment effects were analyzed by mixed model ANOVA with dam as subject and LSD post hoc t-test (***P < .001).

Following the dose response experiment, the moderate dose of 15 µg of clomiphene citrate was utilized for further experiments to investigate the effects of clomiphene citrate exposure on subsequent fetal development. This dose (0.75 mg/kg in a 20 g mouse) is comparable with the lowest dosage of 50 mg of clomiphene citrate commonly prescribed to women (1.0 mg/kg/day in a 50 kg woman). Mated CBA F1 mice were administered 15 µg of clomiphene citrate in the periconception phase on GD 0.5 and GD 1.5, then pregnancy outcomes were assessed on GD 18.5. Pregnancy rate (the proportion of dams with at least 1 implantation site) was 23% less in the clomiphene citrate–treated females than in the control females (Fig. 3A, *P* = .089). Within pregnant dams, no significant effect on total litter size, viable implantations, or resorptions was seen (Fig. 3B-D). Fetal weight was reduced by 16% in clomiphene citrate-treated dams compared with controls (Fig. 3E, *P <* .001). Placental weight was not significantly changed (Fig. 3F), and there was a decrease in fetal:placental weight ratio of 22% (Fig. 3G, *P <* .001). Images of representative fetuses from clomiphene citrate–treated dams shows they were often smaller and had delayed development compared with controls (Fig. 3H).

**Figure 4.**
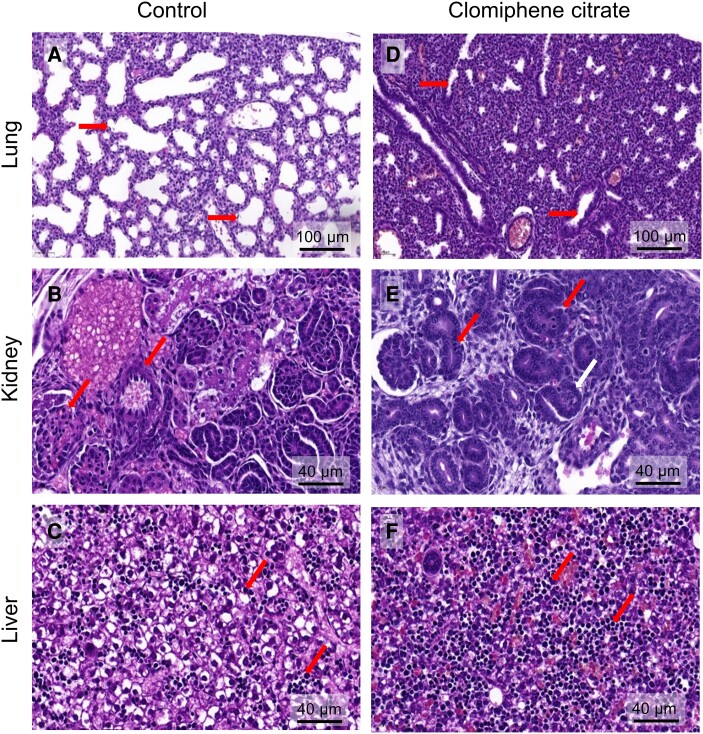
The effects of periconception clomiphene citrate on fetal developmental outcomes at GD 18.5. CBA F1 females were mated to BALB/c males and administered 15 µg of clomiphene citrate or saline control subcutaneously on GD 0.5 and GD 1.5; then fetuses were recovered at GD 18.5, fixed in Bouin's fixative, and processed for fetal developmental and histological analysis at the Australian Phenome Network (University of Melbourne) (see Materials and Methods for details). Panels show histological images from fetuses of control dams (left hand side) and fetuses from clomiphene citrate–treated dams (right hand side) in which developmental anomalies in the lung, kidney and liver were detected (see Table 1). Histology in the lung was representative of several fetuses showing similar delay in lung development.

**Figure 5.**
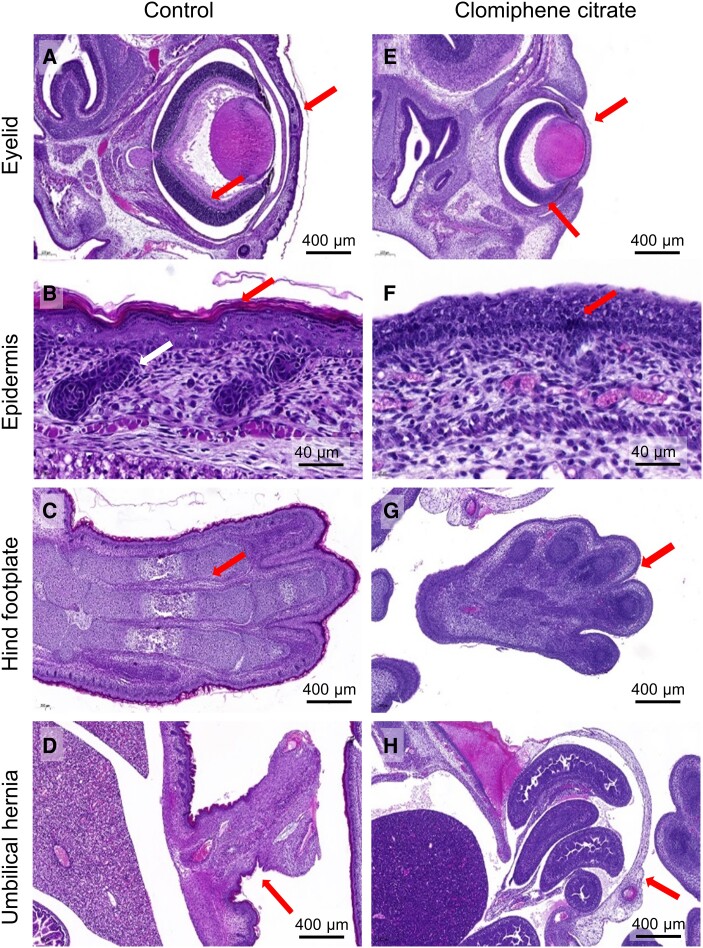
The effects of periconception clomiphene citrate on fetal developmental delay at GD 18.5. CBA F1 females were mated to BALB/c males and administered 15 µg of clomiphene citrate or saline control subcutaneously on GD 0.5 and GD 1.5, then fetuses were recovered at GD 18.5, fixed in Bouin's fixative and processed for fetal developmental and histological analysis at the Australian Phenome Network (University of Melbourne) (see Materials and Methods for details). Panels show histological images from 1 fetus of a control dam (left hand side), and 1 fetus from a clomiphene citrate–treated dam (right hand side) in which developmental delay was detected, and was associated with anomalies in eyelid, epidermis, and hind footplate development, and umbilical hernia regression.

To further evaluate the impact of clomiphene citrate on fetal development, fetuses from a subset of dams were evaluated by morphological and histological analysis to determine the impact of maternal clomiphene citrate exposure. Of 30 fetuses taken from 13 dams given clomiphene citrate, 10 (33%) fetuses from 6 dams showed evidence of delayed development (Table 1). Compared with reference material, the clomiphene citrate-exposed fetuses (CC 1-1, CC 2-1, CC 2-2, CC 87-1, CC 88-1, CC 88-2, CC 88-3, CC 89-1, CC 89-2, and CC 89-4, see Table S1 (32)) showed signs of earlier than expected stages of development. This was notably evident in lung tissue, which exhibited very thick alveolar walls with small or unapparent alveolar spaces (Fig. 4A and 4D), and liver, which exhibited marked extramedullary hemopoiesis (Fig. 4B and 4E). The renal glomeruli were also less developed and more cellular in many of these fetuses (Fig. 4C and 4F), and some fetuses showed remains of umbilical hernia. One fetus (CC 88-1) had developmental features consistent with severely delayed development (TS 22-23, normally expected at GD 14.5-15.5). These features included eyelids not fused; primitive morphology of the retina; substantial physiological umbilical hernia; paddle-like digits, and absence of stratified epidermis (Fig. 5). In addition, 9 fetuses showed evidence of aberrant bleeding including blood in peritoneal cavity in 5 fetuses (16.7%); interstitial hemorrhage in footplate in 1 fetus (3.3%), interstitial hemorrhage in liver in 2 fetuses (5.7%), and subcutaneous hematoma in 1 fetus (3.3%). Whether this was caused by agonal artefact and/or trauma at the time of excision was unclear. No significant morphological changes were evident in the brain, spinal cord, heart, or other tissues. None of the fetuses from control dams showed any features indicating deviation from expected developmental stages and tissue structures.

**Table 1.** Summary of developmental anomalies observed in GD 18.5 fetuses of dams administered clomiphene citrate on GD 0.5 and 1.5

Fetal developmental anomaly*^a^*	Number (sex)	Percentage
Moderate developmental delay	6 F, 3M	30.0% (9/30)
Extensive developmental delay	1 F	3.3% (1/30)
Immature lung development*^b^*	6 F, 3 M	30% (9/30)
Remains of umbilical hernia	7 F, 3M	33% (10/30)
Blood in peritoneal cavity*^c^*	3 F, 2 M	16.7% (5/30)
Interstitial hemorrhage in footplate*^c^*	1 F	3.3% (1/30)
Interstitial hemorrhage in liver*^c^*	2 M	5.7% (2/30)
Subcutaneous hematoma*^c^*	1 F	3.3% (1/30)

Abbreviations: F, female; M, male.

^*a*^Photomicrographs showing representative histological features of each anomaly are shown in Figs. 4 and 5.

^*b*^Fetuses exhibiting immune lung development also commonly showed remains of umbilical hernia, marked extrameduallary hemopoiesis, and immature renal glomeruli.

^*c*^Bleeding may be due to agonal artefact or trauma.

### Clomiphene Citrate Treatment Prolongs Gestational Length and Causes Fetal Loss at Birth

**Figure 6.**
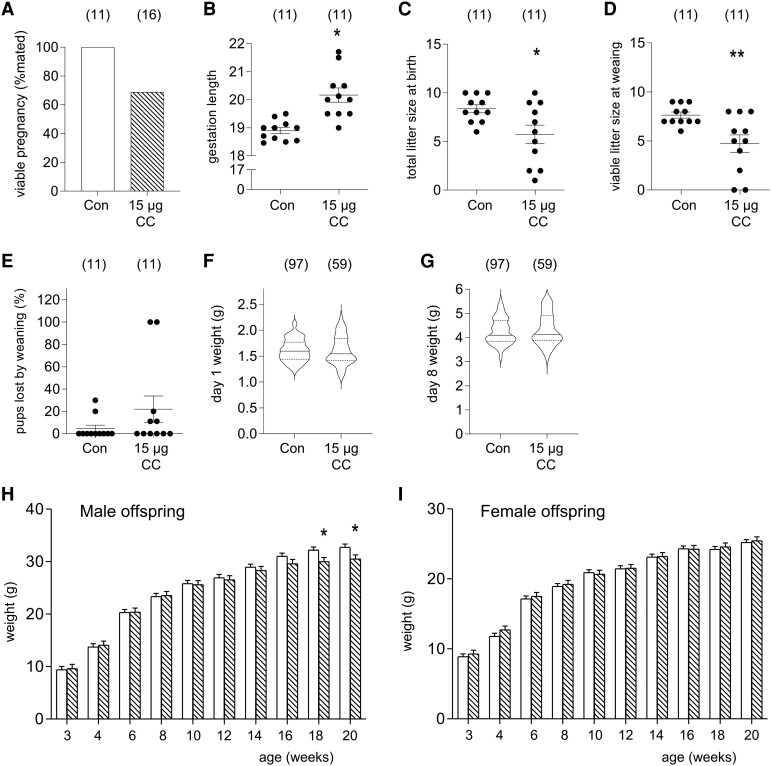
The effects of periconception clomiphene citrate on postnatal outcomes. CBA F1 females were mated to BALB/c males and administered 15 µg of clomiphene citrate or saline control subcutaneously on GD 0.5 and GD 1.5 and allowed to progress to birth. Pregnancy rate is the proportion of mated mice with at least 1 viable pup at birth (A). Other data are mean + SEM gestational length (B), total litter size (C), number of viable pups at weaning (D), percentage of pup loss by 1 week (E). Pups were weighed on day 1 (F) and day 8 (G) after birth, then sexed at 21 days and weighed every 2 weeks until week 20 (H, I). For A-F, the number of mated dams per group is shown in parentheses. Treatment effects were analyzed by χ^2^ test for pregnancy viability and Sidak t-test for other parameters (*P < .05). For F and G, the number of progeny is shown in parentheses. For F-I, data is mean + SEM and treatment effects were evaluated using Mixed Model Linear Repeated Measures ANOVA and post hoc LSD t-test to compare the clomiphene citrate and control groups (*P < .05).

We then evaluated pregnancy outcomes at term and offspring postnatal development in a third cohort of mated CBAF1 mice administered 15 µg of clomiphene citrate or saline in the periconception phase on GD 0.5 and GD 1.5. Pregnancy rate was reduced by 31% in dams given clomiphene citrate compared with controls (Fig. 6A, *P =* .067). For control dams, the mean time of pup delivery was GD 18.9, while dams given clomiphene citrate delivered on average 30 hours later (Fig. 6B, *P =* .002). Litter size at birth was reduced by 32% for dams given clomiphene citrate (Fig. 6C, *P =* .020). By weaning, the number of viable pups remaining was further reduced by 38% in litters of dams given clomiphene citrate (Fig. 6D, *P =* .006). Although a higher percentage of pups were lost between birth and weaning, this difference did not reach statistical significance (Fig. 6E).

There was no effect of clomiphene citrate on offspring weight at postnatal day 1 or postnatal day 8 (Fig. 6F and 6G). Male offspring of clomiphene citrate–treated mothers had similar weights at 3 weeks until 16 weeks of age. At 18 and 20 weeks of age, male offspring of clomiphene citrate-treated dams were significantly lighter than controls (Fig. 6H, 6.9% lighter at 18 weeks, *P =* .035; 6.8% lighter at 20 weeks, *P =* .033). There was no significant difference in weight observed in female offspring of clomiphene citrate–treated dams compared with control dams from 3 weeks until 20 weeks of age (Fig. 6I).

At weaning on 21 days of age, the AGD was measured in both male and female offspring. AGD was increased in both male progeny (*P =* .012) and female progeny (*P <* .001) from dams given clomiphene citrate compared with offspring of control dams (Fig. S2A (32)). Female offspring were assessed daily from 21 days of age for the onset of vaginal opening, but no difference in time of onset was observed between treatment groups (Fig. S2B (32)).

At week 20, male and female offspring were killed and body morphometry analysis was performed to evaluate the effects of clomiphene citrate on individual organs and tissues (Tables S1 and S2 (32)). Male progeny of dams given clomiphene citrate had a 16% smaller retroperitoneal fat mass and 31% smaller perirenal fat mass than male offspring of control dams (Table S1 (32), both *P <* .05), with similar trends in subcutaneous fat and total central fat. A reduction in fat deposition remained evident after fat mass was normalized to body weight. The absolute weight of adrenal glands was reduced by 18% in offspring of dams given clomiphene citrate (Table S1 (32), *P <* .05), but no change was seen after normalization to body weight. No other organs or tissues of male offspring showed changes due to treatment group. There were no changes in organ and tissue weights in female offspring of dams given clomiphene citrate, regardless of whether absolute values or values normalized to body weight are considered (Table S2 (32), *P <* .05). No other organs or tissues were affected by dam treatment. Sperm parameters were also assessed in adult male offspring, and no significant differences were observed (Table S3 (32)).

### Clomiphene Citrate Treatment Attenuates the Uterine Immune Response

**Figure 7.**
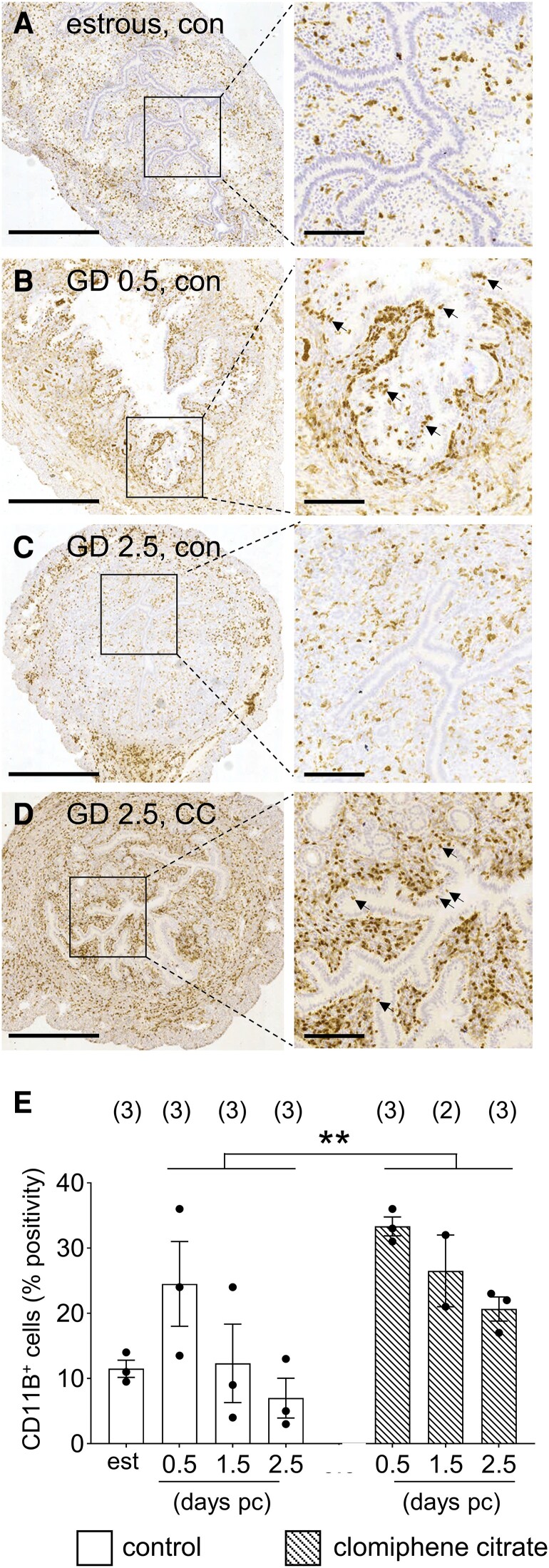
The effects of periconception clomiphene citrate on CD11B^+^ leukocytes (neutrophils and monocyte/macrophages) in the uterus. CBA F1 females were mated to BALB/c males and administered 15 µg of clomiphene citrate or saline control subcutaneously on GD 0.5 and GD 1.5, or were unmated estrous controls. Mice were killed on GD 0.5, GD 1.5, or GD 2.5 (2 hours after most recent clomiphene citrate treatment was administered on GD 0.5 or 1.5). Sections of uterus were analyzed by immunohistochemistry to identify CD11B^+^ cells (brown staining, arrows). Representative photomicrographs of uterus from estrous mice (A), saline-treated mated mice on GD 0.5 (B) and GD 2.5 (C), and clomiphene citrate–treated mice on GD 2.5 (D) are shown, at low power (left-hand side) and high power of boxed region (right-hand side). (E) CD11B staining (% positivity) was quantified by image analysis, and analyzed by 2-way ANOVA to determine the effect of clomiphene citrate treatment. The number of mice per time point/treatment group is shown in parentheses. Bar in left-hand side panels = 1000 µm; Bar in right-hand side panels = 200 µm. **P = .006.

Finally, to investigate the possibility of an effect of clomiphene citrate on endometrial receptivity, we undertook histological examination of the uterine endometrium across the course of the preimplantation phase of early pregnancy. Uterine tissue was recovered from CBAF1 mice administered 50 µg of clomiphene citrate or saline on GD 0.5 and GD 1.5, and tissue was recovered on GD 0.5, GD 1.5, and GD 2.5, and from unmated estrous mice. After preliminary analysis of sections stained with hematoxylin and eosin suggested an altered inflammatory response in the clomiphene citrate group, we utilized immunohistochemistry with anti-CD11B to detect neutrophils and monocyte/macrophages. Both groups of mice showed the expected inflammation-like response to seminal fluid, indicated by elevated CD11B^+^ leukocytes on GD 0.5 compared with the estrous controls (Fig. 7A and 7B). In saline-treated mice the inflammatory response was resolved 48 hours later at GD 2.5, around 24 hours prior to embryo implantation (Fig. 7C). In contrast, treatment with clomiphene citrate disrupted the resolution and caused a persistently elevated inflammatory state, such that the leukocytic infiltrate was still evident on GD 2.5 (Fig. 7D). Notably, cells with characteristic features of neutrophils were seen within and immediately subjacent to the epithelial lining of the uterine lumen in the clomiphene citrate group (Fig. 7D), but were absent from the endometrium of saline controls (Fig. 7C). Image analysis to quantify CD11B staining showed that clomiphene citrate caused elevated CD11B staining (*P* = .006) that was most evident on GD 2.5 (Fig. 7E).

## Discussion

In this study, we report experiments demonstrating a substantial impact on reproductive outcome after administration of clomiphene citrate to mated mice during the 36 hours following conception. Consistently adverse effects were observed in 3 independent cohorts, specifically a reduced incidence of pregnancy, delayed fetal development and fetal growth restriction in late gestation, delayed parturition, and substantially elevated incidence of perinatal death. Reduced pregnancy rate and fetal growth impairment was observed even at the lowest dose of 5 µg (equivalent to 0.25 mg/kg). These findings demonstrate unequivocally that clomiphene citrate acts in the preimplantation phase to exert harmful effects on fetal survival and development in mice. The data raise the concerning prospect that similar adverse effects of clomiphene citrate exposure might occur in humans.

The moderate and high doses of clomiphene citrate used in this paper correlate to clinical doses of 50 to 100 mg (equivalent to 1-2 mg/kg, in a 50 kg woman), which are the recommended starting dose, and first incremental dose increase, respectively, for fertility treatment in women (33). The manufacturer recommends further increases in dose up to 150 and 200 mg per day, when clinically warranted. Clomiphene citrate consists of a mixture of 3:2 ratio of 2 stereoisomers—enclomiphene and zuclomiphene. While enclomiphene has a relatively short biological half-life of 5-7 days, zuclomiphene persists at high levels in peripheral blood for at least 4 weeks after ingestion (34, 35) and remains detectable up to 6 weeks later (36). It is found to accumulate in women over the 5 day course and across consecutive treatment cycles, potentially remaining in the blood at a significant level for 3 to 4 weeks (37). The recommended treatment protocol is designed to allow a “washout” period of several days between cessation of drug administration and the likely time of conception. However, unsupervised use of clomiphene citrate, as well as the evidence of drug accumulation and persistence through a repeated dose regimen, suggest that early embryogenesis may ensue in many women who conceive after using the drug while substantial levels of clomiphene citrate remain in circulation. The levels in key specific tissues such as the ovary or uterus is unknown, and may be much higher if these tissues selectively accumulate these compounds.

We acknowledge that the treatment schedule used in this study does not fully recapitulate the protocol used in women. Although the doses we gave to mated mice are comparable after adjustment for body weight, women following the recommended schedule will usually have ingested a higher amount over the 5 days of treatment in a given cycle, and will usually have commenced dosing earlier in the cycle, prior to ovulation. We opted to administer clomiphene citrate after ovulation and mating to separate the effects of clomiphene citrate in the ovary from the effects in the endometrium, to mimic effects in the endometrium that would occur in women due to residual accumulated drug. Despite the 2 day vs 5 day dosing schedule, and the fact that many women will accumulate drug over several cycles and will take a higher dose than the recommended starting dose, it is possible that the mice were exposed to a higher concentration of drug in early pregnancy than usually occurs in women because of the altered administration timing. Nevertheless, we contend our experimental protocol is valid for the research question we posed, and note that adverse effects were seen even at the lowest dose we evaluated.

The pregnancy parameters we found to be impacted by clomiphene treatment are consistent with effects from early pregnancy onwards. These effects appear to commence prior to or shortly after embryo implantation, as many female mice administered clomiphene citrate showed no sign at autopsy of having initiating pregnancy. There was a substantial reduction in the proportion of mated mice with viable fetuses or neonates upon analysis at GD 14.5, GD 18.5, or following the expected time of birth. When data from all 3 cohorts of mice administered the moderate dose of 15 µg were combined, pregnancy rate was reduced by 30%, from 32/34 (94%) in control mice, to 32/49 (65%) in mice administered clomiphene citrate (*P* = .004).

There has been only limited evaluation of effects of clomiphene citrate in in vivo preclinical models. Several studies in rats around the time of drug development point to impaired implantation after clomiphene administration in the periovulatory phase or immediately after mating (28, 38-40). In 1 previous study clomiphene citrate was administered to mice in the periconception phase, at a range of preconception and postconception time points (27). This study showed a substantial adverse impact on embryo implantation rates particularly when compound was administered at times corresponding to ovulation or early after conception. That the effect was at least partly mediated by reduced uterine receptivity to embryo implantation was demonstrated by embryo transfer experiments (27). Consistent with an endometrial effect, a second study used blastocyst transfer to demonstrate that preimplantation administration of clomiphene citrate (at a high dose of 50 µg/day on days 0.5-2.5 post coitum) caused extensive implantation failure in mice (41). Similar effects on implantation attributed to endometrial receptivity have been reported in rabbits (42). However, whether reduced oocyte quality, ovulation failure, and/or deficits in early embryo development exerted by clomiphene citrate contribute to impaired fertility in animal studies has not been clearly determined.

The current study adds to these findings by providing clear evidence that the mechanisms by which clomiphene acts in the periconception environment are not constrained to effects in the oocyte imparted prior to ovulation. Given that we did not administer compound until after ovulation, mating, and fertilization, an effect on uterine receptivity to implantation and early placental development seemed the most likely mechanism for the outcomes we observed, although effects on early embryo development could also contribute. Because of its estrogenic and antiestrogenic properties, clomiphene has potential to impact also the uterine and oviductal secretions and microenvironment, so epigenetic or developmental modifications affecting preimplantation embryo development are possible.

Consistent with an effect mediated via the female reproductive tract, we found that clomiphene citrate profoundly perturbed the uterine immune environment. Remarkably, neutrophils that normally disappear within 24-36 hours of mating remained abundant on GD 2.5, and excessive CD11B^+^ macrophages were also apparent. Given the dynamics of this immune response has a major bearing on endometrial receptivity (43), and acquisition of receptivity commences well before embryos adhesion and trophoblast invasion (44), it seems likely that persistent inflammation at this time could impact the capacity of the endometrium to undergo a decidual response and support implantation. Further experiments are warranted to more fully evaluate the effect of clomiphene citrate on endometrial receptivity and the immune response to pregnancy, and the potential for a mechanism involving these events to affect fetal development.

The second most notable impact we found was impaired fetal development in those dams where pregnancy did progress. The moderate 15 µg dose of clomiphene citrate treatment acted to reduce fetal weight and length, and indicators of fetal developmental stage showed developmental delay, measured in Theiler scores assigned at GD 14.5 and GD 18.5. Histological analysis at GD 18.5 showed compelling evidence of developmental anomalies, with close to a third of all fetuses exhibiting structural features of the lung, liver, kidney, and umbilicus more consistent with earlier phases of development than expected at GD 18.5. Fetal growth restriction most often occurs as the result of altered placental development causing impaired placental transport function (45). Although placental size (in viable implantation sites) was not affected by clomiphene exposure, constrained placental function can occur without gross morphological changes and a smaller fetal:placental weight ratio supports the likelihood of reduced placental functional efficiency (46). Placental development is strongly influenced by the decidual response and endometrial immune parameters in the implantation phase (47-49), so fetal developmental perturbations could well be a consequence of the same pathophysiological mechanism underlying the observed implantation failure.

Direct effects of clomiphene citrate acting in the placenta and fetus may also contribute to fetal developmental anomalies. As well as features indicating developmental delay, histological analysis revealed evidence of hemorrhage and/or hematoma in many fetuses, most often in the peritoneal cavity or interstitial spaces of skin or pelvic organs, suggesting a potential vascular defect. The mechanisms causing placental defects as well as implantation failure may involve the canonical estrogen receptor interaction and/or off-target actions of clomiphene citrate. It is relevant that triphenylethylene antiestrogens have been linked with defects in angiogenesis and vascular endothelial growth factor signaling in several tissues including the uterus, lung, and eye (50-53). In women, the eye is particularly sensitive to the effects of clomiphene where visual disturbances are a common side-effect (54, 55). Effects of clomiphene citrate on the vasculature of the fetus and/or placenta could exacerbate many of the observed developmental defects.

Surprisingly, we found clomiphene citrate treatment acted to prolong gestation length by 30 hours, a substantial delay given the short duration of pregnancy in mice. This could be secondary to the adverse effects on fetal growth and maturation, as cues derived from the mature fetal lung are involved in triggering the parturition cascade in mice (56). To our knowledge, only 1 recent study has reported delayed parturition, in rats given 2.0 mg/kg clomiphene citrate in midgestation (28). When pups were weighed at 12 to 24 hours after birth, there was no difference between pups of clomiphene citrate-treated dams and pups of control dams. This contrasts with the fetal weight difference at GD 18.5, consistent with an altered trajectory of fetal growth in the days prior to birth and suggesting that clomiphene citrate-exposed pups “catch up” to control pups during their extended gestation prior to birth. This “catch up” might be facilitated by a smaller viable litter size in this group, but likely occurs independently of litter size as several clomiphene-treated dams sustained large litters to birth. Therefore, it is reasonable to conclude that the growth trajectories of fetuses exposed to clomiphene citrate deviate from the normal pattern.

An additional impact of clomiphene exposure was late gestation fetal death and/or early postnatal pup death. This is evident in the difference in litter size observed between the GD 18.5 and the postnatal cohort, and implies that many of the developmentally impaired fetuses in the litters of clomiphene citrate–treated dams did not survive the perinatal transition. These pups are likely to be delivered and then die shortly after birth, but this is difficult to accurately record in mice due to maternal cannibalization of pups. In contrast, there were comparable numbers of pups in litters of control dams measured at late gestation on GD 18.5, and at birth.

Two previous studies of clomiphene citrate administration during early pregnancy in mice documented both fetal growth restriction and teratogenic effects (26, 27). Dziadek et al (27) reported fetal congenital anomalies, including exencephaly and hydrocephaly, associated with reduced postnatal survival. Ara and Asmatullah (26) administered clomiphene citrate (at doses of 1.0-6.0 mg/kg) to pregnant mice on day 8 of gestation, and reported substantial fetal loss and fetal growth restriction evident as decreased fetal weight, crown rump length, head circumference, and other measures. In a comprehensive morphological assessment, this study also reported developmental anomalies at day 18 of gestation in many fetuses, including open eyelids, anophthalmia, fore and hindlimb micromelia, meromelia, amelia, sacral hygroma, hydrocephaly, hemorrhagic spots, kyphosis, and clubbed feet. These findings were supported by histological assessment of fetuses revealing extensive malformations in many tissues (26). The fetal growth restriction in the 2 prior studies is broadly consistent with the decreased fetal weight and delayed fetal development we observed at both GD 14.5 and GD 18.5, with lower doses than the previous studies, but on balance the current study does not recapitulate the same degree of congenital abnormality seen previously. In particular, our histological analysis of fetuses did not reveal brain or neural defects, or head or limb anomalies. Whether this is due to the higher dose and/or later timing of compound administration in the earlier studies, or strain of mouse used, remains to be investigated. It is also possible that our limited analyses underestimated the extent of developmental disruption—indeed this would be consistent with the high degree of perinatal loss we observed.

In broad terms, we found those pups that survive the first days of life following birth after in utero exposure to clomiphene citrate developed into adulthood with a relatively normal growth and developmental trajectory. Nevertheless, we found indications of alterations to reproductive tract development that are consistent with endocrine-disrupting effects, and reminiscent of links between clomiphene citrate exposure and risk of hypospadias and related urogenital defects in exposed infants (22). Anogenital distance is a common marker of endocrine disruption and potential for subsequent testicular dysfunction, by virtue of association with serum testosterone levels in animal models (57, 58). The increased anogenital distance observed in both male and female progeny from clomiphene citrate–treated dams in the current study might reflect increased testosterone levels (59), consistent with clinical reports that clomiphene increases circulating testosterone (60). The reduction in fat deposition we observed in male progeny of clomiphene citrate–treated dams is also consistent with an endocrine and/or metabolic perturbation (61). Previous studies in rats administered 2.0 mg/kg clomiphene citrate shortly after implantation on GD 5 showed multiple abnormalities of reproductive tracts in adult female offspring, including disorganized hyperplastic epithelium in the vagina and uterus, consistent with long-acting estrogenic effects and hyperestrogenization of fetal tissues (28). Different effects on offspring reproductive tract tissues might be seen depending on the timing and dose of clomiphene exposure.

It is relevant to consider the degree to which these findings are relevant to clomiphene citrate effects in women. There are limitations to which mouse data can be extrapolated to human, because of the obvious genetic and physiological differences between species, especially in regard to reproductive strategy and anatomy (62, 63). Another limitation is the difference in administration protocols—we intentionally delivered compound after ovulation to exclude effects on oocytes, while in women, clomiphene is delivered earlier in the reproductive cycle, and often over multiple cycles (33, 64). Because in women clomiphene administration commences in the proliferative phase in order to promote ovulation, it is difficult to discern whether an increased conception rate may mask a reduced implantation rate.

Several studies have investigated the effects of clomiphene citrate on endometrial biology and function, and a range of effects including reduced endometrial thickness and uterine volume are reported in a subset of women, and are associated with elevated miscarriage rates (65-68). In women with poor endometrial growth, clomiphene elicits cellular and molecular effects including aberrant expression of estrogen receptor, altered gene and protein expression suggesting reduced proliferation and angiogenesis, and—similar to the mouse findings herein—elevated inflammation indicated by leukocyte accumulation and aberrant expression of immune-regulatory genes (69). Others argue that high miscarriage rates in women using clomiphene citrate is due to poor quality oocytes (70). The extent to which underlying conditions associated with subfertility may be causal, vs effects of the drug, has also been debated (17, 36). Arguing against this, there is compelling evidence of risks for women using clomiphene citrate including elevated incidence of endometrial and ovarian cancer at higher doses or with prolonged use (10, 12, 71-74).

Congenital defects such as cleft palate, hypospadias, hydrocephaly, septal heart defects, neural tube defects, and blindness are among the list of birth defects reported in studies of infants born after maternal clomiphene citrate treatment (22, 74, 75). A study in a Danish cohort reports elevated leukemia and central nervous system–related tumors in children conceived after maternal clomiphene citrate treatment (76). Aberrant vascular development in the fetus and/or placenta, as well as maternal endometrial effects, may contribute to these developmental anomalies (17, 50-53).

Despite the many studies on clomiphene citrate since its introduction as a breakthrough infertility treatment, there has been no longitudinal study of safety, and the available studies are limited by study design often resulting in inconclusive findings (14, 17). The wide range of effects of triphenylethylenes in ovulation, pregnancy, and fetal development strongly suggest further analysis is required to consider safety, timing and dose effects, and maternal susceptibility factors including drug metabolism and factors related to subfertility.

In summary, the observations here of adverse pregnancy outcomes after clomiphene citrate administration mirror those observed in previous animal studies and humans (17). The current study adds to the evidence that clomiphene citrate is sufficient to cause adverse events, independently of fertility status, and indicates biological plausibility for a comparable effect in humans. Our findings therefore strongly support calls for additional investigation of the use of clomiphene citrate in women, and indicate that conservative supervision and dosing to reduce the level of fetal exposure is warranted.

## Data Availability

Some or all datasets generated during and/or analyzed during the current study are not publicly available but are available from the corresponding author on reasonable request.

## Abbreviations

AGD: anogenital distance
ANOVA: analysis of variance
CC: clomiphene citrate-exposed fetuses
DAB: 3,3'-diaminobezidine
GD: gestational day
SEM: standard error of the mean
TS: Theiler stage
